# Direct-acting antivirals and visceral leishmaniasis: a case report

**DOI:** 10.1186/s12879-019-3947-x

**Published:** 2019-04-18

**Authors:** Claudia Colomba, Laura Saporito, Paola Di Carlo, Manlio Tolomeo, Adriana Cervo, Alberto Firenze, Marcello Trizzino, Antonio Cascio

**Affiliations:** Dipartimento di Scienze per la promozione della Salute e Materno-infantile, Università di Palermo, Palermo, Italy

**Keywords:** Hepatitis C, Leishmania, Visceral Leishmaniasis, Direct-acting antiviral

## Abstract

**Background:**

Visceral leishmaniasis is a vector-borne parasitic disease caused by protozoa belonging to the genus *Leishmania*. The clinical presentation of visceral leishmaniasis strictly depends on the host immunocompetency, whereas depressive conditions of the immune system impair the capability to resolve the infection and allow reactivation from sites of latency of the parasite.

**Case presentation:**

We describe a case of visceral leishmaniasis (VL) that occurred in a patient with chronic hepatitis C treated with direct-acting antiviral drugs (DAA). The hypothesized mechanism is the alteration of protective inflammation mechanisms secondary to DAA therapy. Downregulation of type II and III IFNs, their receptors, which accompany HCV clearance achieved during treatment with sofosbuvir and ribavirin might have a negative impact on a risk for reactivation of a previous *Leishmania* infection. We know indeed that IFN-γ is important to enhance killing mechanisms in macrophages, which are the primary target cells of *Leishmania*.

**Conclusion:**

Since VL is endemic in Sicily as well as in other countries of the Mediterranean basin, physicians should be aware of the possible unmasking of cryptic *Leishmania* infection by DAAs.

## Background

Visceral leishmaniasis (VL) is a vector-borne parasitic disease caused by protozoa belonging to the genus *Leishmania*. The clinical presentation of visceral leishmaniasis strictly depends on the host immunocompetency and ranges from asymptomatic to severe forms characterized by fever, splenomegaly and pancytopenia. Depressive conditions of the immune system such as HIV infection, immunosuppressive treatments, and cirrhosis impair the capability of the immune response to resolve the infection and allow reactivation from sites of latency of the parasite [1–4]. VL has also been reported during pegylated interferon therapy for chronic hepatitis C [5]. Over 70 million people worldwide are chronically infected with hepatitis C virus (HCV) and it is estimated that in Sicily there is a burden of at least 150,000 HCV infected patients: since 2015 almost 10,000 patients were treated with direct-acting antiviral agents (DAA) [6]. IFN-free DAA combination therapies for HCV are oral regimens that are well tolerated, and typically induce rapid and sustained viral response (SVR) [7]. Anyway the effect of DAA on the incidence of other infections in clinical practice is still unclear. We describe here a case of VL that occurred in a patient with chronic hepatitis C treated with DAA.

## Case presentation

A Sicilian seventy-year-old man was admitted to the Infectious Diseases Unit of the University Hospital of Palermo (Sicily, Italy) in July 2017 referring progressive asthenia and irregular fever not responsive to antibiotic therapy lasting 2 months. The patient suffered from diabetes mellitus, mild kidney disease, and HCV-related liver cirrhosis (Child-Pugh B7), for which had been treated with sofosbuvir and ledipasvir for 24 weeks until December 2015.

About 3 months later the end of the HCV-treatment, the patient suffered from a marked worsening of thrombocytopenia, not considered related to liver cirrhosis and not responding to steroid therapy for which he had been treated with romiplostim whenever platelet count was under 20,000/mmc.

At admission in our division, the patient had fever (BT 37.8°) with a pulse rate of 78 bpm, blood pressure and respiratory rate were normal. No other symptoms were reported except asthenia. At the physical examination he had no respiratory alterations, systolic heart murmur at apex (grade II/VI), hepatosplenomegaly and grade 1-encephalopathy with bradilalia and minimal flapping tremor.

Laboratory tests revealed pancitopenia (WBC 3100/mmc -neutrophils 46%, lymphocytes 33%-, Hb 8.1 g/dl, platelets 46,000/mmc), an increased C-reactive protein (33 mg/l), D-dimer 6980 UI/l and INR 1.5, eGFR 63 ml/min/1.73m^2^, AST/ALT 2xN, modest hypoalbuminemia with polyclonal gammopathy.

The abdominal ultrasound exam showed an increase in splenomegaly (17 cm) compared to the previous ultrasonography from 1 month earlier (14.5 cm). Thoracic CT scanning was normal. Trans-thoracic echocardiography excluded vegetations. Blood culture, brucellosis serology, HIV-IgG and HBsAg were negative. *Leishmania* serology resulted positive in enzyme-linked-immunosorbent-assay (IgG 1:12800), immunofluorescent antibody test (IgG 1:25600) performed as previously described [2] (Table 1) and Western Blot.

**Table 1:** Serological tests indicating *L. infantum* infection

*L. infantum* diagnostic test	Positivity threshold	Patient’s value
Enzyme-linked-immunosorbent-assay (ELISA) (IgG)	1:50	1:12800
Immunofluorescent antibody test (IFAT) (IgG)	1:50	1:25600

*Leishmania infantum* DNA was detected in peripheral blood by Real-time Polymerase Chain Reaction (PCR) targeted on a 117 bp fragment in the mini circle kinetoplast DNA (kDNA), carried out as previously described by Vitale et al. [8].

A bone marrow aspirate was not performed because of low platelet count. Liposomal amphotericin B was started at the dose of 3 mg/kg from day 1 to 5 and a further dose on day 10 for a total dose of 18 mg/kg.

The blood cells count and the clinical conditions of the patient progressively improved until reaching normal values; *L. infantum* real-time PCR on peripheral blood at the end of the treatment was negative.

## Discussion and conclusions

It is well known that T-cell-dependent immune response and macrophage activation play a major role during primary *Leishmania* infection or reactivation of latent infection, in addition the effect of DAA on the host immune status has also recently been addressed [1, 7]. It has been well documented that DAA regimens are associated to lower side-effects and higher rate of SVR obtained in a shorter time frame than interferon and ribavirin. However, some researchers are now discovering an increased rate of de novo and recurrent hepatocellular carcinoma (HCC) in patients with HCV cirrhosis compared to interferon treatment protocols. The hypothesized mechanisms is that inflammation secondary to chronic HCV infection plays a role in immunosurveillance. DAA therapy seems to abolish this protective and antineoplastic effect [9]. Moreover, Meissner et al. demonstrated that treatment with sofosbuvir and ribavirin is associated to downregulation of type II and III IFNs, their receptors and interferon stimulated genes in liver [10]. This might have a negative impact on immune control allowing reactivation of a previous infection. HBV reactivation occurs frequently in patients with chronic HBV and HCV coinfection receiving DAA therapy. It is therefore important to have all patients screened for evidence of overt or occult HBV infection and properly manage the coinfection during DAAs therapy [11].

We know that IFN-γ is important to enhance killing mechanisms in macrophages, which are the primary target cells of *Leishmania* as well as other intracellular infections such as tuberculosis. Consequently, a potential risk for reactivation of a previous *Leishmania* or *Mycobacterium tuberculosis* infection during DAA therapy is possible [12]. Since VL is endemic in Sicily as well as in other countries of the Mediterranean basin, and physicians should be aware of the possible unmasking of cryptic *Leishmania* infection after DAAs therapy. Even if this risk is not considered as a contraindication for anti-HCV treatment, baseline screening could be suggested when this treatment is started in these epidemiological settings.

## Abbreviations

bpm: beats per minute
BT: Body temperature
CT: Computed tomography
DAA: Direct-Acting Antiviral
eGFR: estimated glomerular filtration rate
Hb: Haemoglobin
HBV: Hepatitis B Virus
HCV: Hepatitis C Virus
HIV: Human Immunodeficiency Virus
IFN: Interferon
PCR: Polymerase chain reaction
VL: Visceral leishmaniasis
WBC: White blood cells
